# Supplementary material for: Altered Gut Microbiome Composition in Dogs with Hyperadrenocorticism: Key Bacterial Genera Analysis
Source: Animals (Basel). 2024 Oct 7;14(19):2883. doi: 10.3390/ani14192883 (PMC11476382; doi:10.3390/ani14192883)
Supplement: Supplementary file 1 [file animals-14-02883-s001.zip › animals-3200921-supplementary.docx]

| **Table S1.** Diet of healthy control and dogs with HAC | |
| --- | --- |
| **Healthy dogs (n=9)** | **HAC dogs (n=15)** |
| **Prescription diet** | |
| Dry food, Royal Canin, Hypoallergenic (n=3) | Dry food, Royal Canin, Hypoallergenic (n=2) |
|  | Dry food, Royal Canin, Hepatic (n=1) |
|  | Wet food, Royal Canin, Gastrointestinal low fat (n=1) |
|  | Dry food, Royal Canin, Gastrointestinal low fat (n=2) |
|  | Dry food, Hills, z/d (n=1) |
|  | Dry food, Hills, k/d (n=1) |
| **Commercial diet** | |
| Dry food, Royal Canin, Mini indoor adult (n=2) | Dry food, other companies, animal based (n=7) |
| Dry food, Royal Canin, Light weight (n=1) |  |
| Dry food, Royal Canin, Pomeranian (n=1) |  |
| Dry food, Royal Canin, Senior (n=1) |  |
| Dry food, other companies, animal based (n=1) |  |
| HAC, hyperadrenocorticism |  |

| **Table S2.** Alpha diversity analyzed using 16S ribosomal ribonucleic acid sequencing in healthy dogs and dogs with HAC | | | |
| --- | --- | --- | --- |
| **Alpha diversity** | **HAC dogs (n=15)** | **Healthy dogs (n=9)** | p-value |
| Chao 1 | 44(34.00, 47.00) | 35(31.50, 40.00) | 0.086 |
| Shannon index | 1.410(1.220, 1.990) | 2.050(1.850, 2.345) | 0.0148* |
| Variables are expressed as median (interquartile range) | | | |
| HAC, hyperadrenocorticism | |  |  |
| *p <0.05, statistically significant differences based on Mann–Whitney test. | | | |

| **Table S3.** Alpha diversity analyzed using 16S ribosomal ribonucleic acid sequencing in dogs with HAC before and after trilostane treatment | | | |
| --- | --- | --- | --- |
| **Alpha diversity** | **HAC dogs** **(n=5)** | | p-value |
|  | **Pre-treatment** | **Post-treatment** |  |
| Chao 1 | 39.00(34.50, 46.00) | 39.00(33.00, 46.50) | 0.875 |
| Shannon index | 1.890(1.300, 2.155) | 1.440(1.305, 2.050) | 0.8125 |
| Variables are expressed as median (interquartile range) | | | |
| HAC, hyperadrenocorticism | |  |  |
| *p <0.05, statistically significant differences based on Wilcoxon signed-rank test. | | | |
